# 20(S)-protopanaxadiol regio-selectively targets androgen receptor: anticancer effects in castration-resistant prostate tumors

**DOI:** 10.18632/oncotarget.24695

**Published:** 2018-04-20

**Authors:** Mohamed Ben-Eltriki, Subrata Deb, Mohamed Hassona, Gray Meckling, Ladan Fazli, Mei Yieng Chin, Nada Lallous, Takeshi Yamazaki, William Jia, Paul S. Rennie, Artem Cherkasov, Emma S. Tomlinson Guns

**Affiliations:** ^1^ The Vancouver Prostate Centre at Vancouver General Hospital, Vancouver, BC, Canada; ^2^ Department of Experimental Medicine, Faculty of Medicine, University of British Columbia, Vancouver, BC, Canada; ^3^ Department of Urologic Sciences, Faculty of Medicine, University of British Columbia, Vancouver, BC, Canada; ^4^ Department of Surgery and Brain Research Centre, University of British Columbia, Vancouver, BC, Canada; ^5^ Department of Pharmaceutical Sciences, College of Pharmacy, Larkin University, Miami, FL, USA

**Keywords:** 20(S)-protopanaxadiol ginsenoside, androgen receptor, apoptosis, castration resistant prostate cancer

## Abstract

We have explored the effects of 20(S)-protopanaxadiol (aPPD), a naturally derived ginsenoside, against androgen receptor (AR) positive castration resistant prostate cancer (CRPC) xenograft tumors and have examined its interactions with AR. *In silico* docking studies for aPPD binding to AR, alongside transactivation bioassays and *in vivo* efficacy studies were carried out in the castration-resistant C4-2 xenograft model. Immunohistochemical (IHC) and Western blot analyses followed by evaluation of AR, apoptotic, cell cycle and proliferative markers in excised tumors was performed. The growth of established CRPC tumors was inhibited by 53% with aPPD and a corresponding decrease in serum PSA was seen compared to controls. The IHC data revealed that Ki-67 was significantly lower for aPPD treated tumors and was associated with elevated p21 and cleaved caspase-3 expression, compared to vehicle treatment. Furthermore, aPPD decreased AR protein expression in xenograft tumors, while significantly upregulating p27 and Bax protein levels. *In vitro* data supporting this suggests that aPPD binds to and significantly inhibits the N-terminal or the DNA binding domains of AR. The AR androgen binding site docking score for androgen (dihydrotestosterone) was −11.1, while that of aPPD was −7.1. The novel findings described herein indicate aPPD potently inhibits PCa *in vivo* partly via inhibition of a site on the AR N-terminal domain. This manifested as cell cycle arrest and concurrent induction of apoptosis via an increase in Bax, cleaved-caspase-3, p27 and p21 expression.

## HIGHLIGHTS

- 20(S)-protopanaxadiol (aPPD) inhibits tumor growth and concurrently induces apoptosis in castrated resistant prostate cancer
- The downregulation of androgen receptor expression is a major mechanism of aPPD anti-proliferating effect
- aPPD leads to elevated levels of p27 and p21 and therefore cell cycle arrest
- N-terminal domain inhibition of androgen receptor by aPPD is proposed as one of the anticancer mechanisms

## INTRODUCTION

Prostate cancer (PCa) is one of the most frequently diagnosed cancers among men. Despite the substantial progress made during the past two decades, PCa remains the third leading cause of cancer death among men in North America and accounts for about 10% of all lethal cancers [1]. To date, therapeutic options for advanced stage PCa are limited. Since the androgen receptor (AR) continues to drive tumour growth, the current treatments include AR antagonists. These drugs are often used in combination with luteinizing hormone-releasing hormone/gonadotropin-releasing hormone agonists and antagonists aiming to shut-down pituitary axis regulated gonadal steroid production. More recently the use of steroidogenesis inhibitors has been designed to combat local intra-tumoural suppression of steroidogenesis and typically they are introduced for the treatment when the disease has progressed to castration resistant PCa (CRPC). Targeted therapies and agents with growth inhibitory properties that work independent of the androgen pathways are of current interest. Novel anticancer compounds derived from natural products present an attractive alternative to synthetic compounds, based on their favorable safety and effectiveness profiles.

Ginseng is one of the top selling natural products in North America and widely used in complementary and alternative medicine worldwide. We have identified a class of naturally derived ginsenoside molecules that target key cell signaling pathways involving the AR and steroidogenesis (known to be dysregulated in PCa) while enhancing vitamin D receptor expression [2]. Ginsenosides are the main pharmacologically active constituents of ginseng, which are triterpenoid saponins with steroid glycosides consisting of a dammarane skeleton attached to one or more sugar moieties [3, 4]. Ginsenosides are primarily classified into two major categories (differential non-sugar structure in the aglycones), namely, 20(*S*)-protopanaxadiol (aPPD) type (e.g. Rbl, Rb2, Rc, Rd, Rg3, and Rh2) and 20*(S)*-protopanaxatriol (aPPT) type (e.g. Re, Rf, Rgl and Rh1) [3–5]. Respective aglycones, such as aPPD from Rh2, are formed through intestinal bacteria-mediated deglycosylation of ginsenosides in gastric acid [6, 7]. The main pharmacologically active constituent of ginseng is aPPD which possess anticancer, antioxidant, antidepressant, anti-inflammatory, and neuroprotective effects in preclinical and clinical studies. By virtue of their multiple targets, it is not surprising that ginsenosides have highly pleiotropic therapeutic activities and are of current clinical relevance. A series of ginsenoside analogs, which are structurally based on 20(s)-protopanaxadiol (aPPD) (known as drug entity S111 in China), has already been used as antidepressants in humans in China and has been synthesized through a combinatorial chemistry approach developed by the Shanghai Innovative Research Centre of Traditional Chinese Medicine (SIRC) [8]. The most bioavailable and potent ginsenoside metabolite, aPPD, has demonstrated anticancer properties in preclinical and human *in vitro* models, including breast cancer, leukemia, intestinal and prostate cancer [9–15]. The fact that aPPD exhibited good efficacy in inhibiting PCa growth and progression, highlights the potential of aPPD in PCa prevention and/or therapy [2, 16, 17].

Preclinical pharmacokinetic studies from our laboratory have demonstrated that ginsenosides can reach to the mouse xenograft prostate tumor site following oral dosing [12, 18]. Following administration of aPPD oral gavage containing ethanol, propylene glycol, and water formulation, aPPD is readily absorbed and is distributed to the key target tissues including tumors [12, 19]. We have shown that *in vitro* aPPD can induce apoptosis and cell cycle arrest, in PCa cells and can inhibit PCa xenograft growth in preclinical mice models [2]. Recently, we have shown that aPPD inhibited growth and induced apoptosis in androgen-dependent PCa cell lines (LNCaP and C4-2) *in vitro*. Administration of aPPD reduced the AR protein levels not only in LNCaP cells but also in C4-2 cells [2]. In addition, aPPD also suppressed the growth of androgen-independent PC-3 prostate xenograft tumors [12]. It has been shown that aPPD acted additively or synergistically when combined with calcitriol *in vitro* as well as with other chemotherapeutic drugs such as docetaxel or paclitaxel to reduce tumor size in human PCa mouse xenograft models [2, 18].

**Figure 1:**
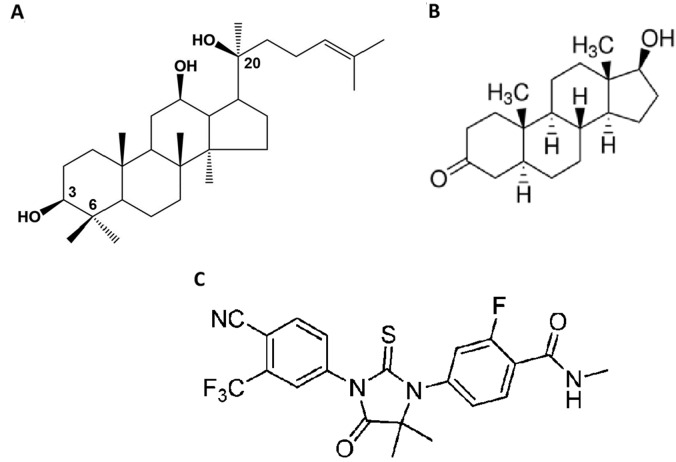
Chemical structure of 20(S)-protopanaxadiol (PPD) **(A)**. Dihydrotestosterone (DHT) **(B)** and enzalutamide **(C)**.

AR is a major driving force in the development and progression of PCa to the metastatic stage and expression of AR splice variants is one of the major mechanisms of CRPC [20]. Androgens binding to AR induces receptor dimerization, which is an absolute requirement for AR signaling [21]. After dimerization, the AR interacts with the DNA-binding domain facilitating DNA binding and the recruitment of cofactors and transcriptional machinery to regulate expression of target genes [21]. AR interaction also exists between an amino terminal domain and ligand-binding domain known as the N-terminal/C-terminal interaction, and ligand-binding domain dimerization. This N/C interaction is an essential factor in regulation of AR activity [21]. Since aPPD exhibited good efficacy in inhibiting AR and its splice variants, this highlights the potential of aPPD in PCa prevention and/or therapy [16, 17]. The aPPD bears structural similarity to androgens that are bound in the AR androgen binding site (ABS) (Figure 1). Previously we have shown that the binding affinity of aPPD to AR is ~10,000-40,000-fold less than dihydrotestosterone (DHT), and it is unlikely that aPPD competes with DHT [16].

The present study is designed to determine if aPPD can inhibit AR-positive castration-resistant C4-2 xenograft prostate tumors. We have also examined and validated potential mechanisms of aPPD-mediated anticancer effects by investigating AR protein expression in tumors, and carried out in *silico* analyses to determine aPPD binding to different domains on the AR as well as *in vitro* assays to determine the ability of aPPD to inhibit AR transactivation. In addition, the effect of aPPD on apoptosis markers (Bax, cleaved-caspase 3), and proliferation markers (ki67) expressions were examined.

## RESULTS

### aPPD inhibits growth of castration-resistant C4-2 tumors in nude mice

**Figure 2:**
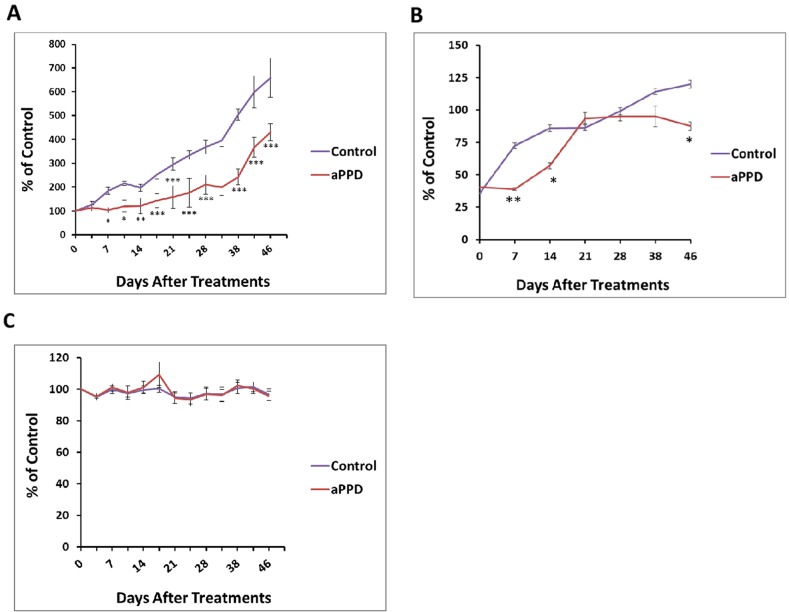
The *in vivo* effect of aPPD on the tumor volume **(A)**, and serum PSA **(B)**. Change in tumor volume was followed over time for mice treated orally with either control (ethanol: propylene glycol: water (2:7:1)) or aPPD (70 mg/kg once daily) formulations. Average tumor volumes are expressed as a percentage of the average initial tumor volume of each week, post C4-2 cells inoculation and castration. *In vivo* toxicity as assessed by change in mean body weight of C4-2 mice xenograft expressed as % of control **(C)**. No animals showed any signs of toxicity or weight loss. Data are presented as Mean value ± SEM, n of 8 in each group. A *p* value < 0.05 was considered significant (^*^), A *p* value < 0.01 was considered very significant (^**^) and a *p* value < 0.001 was considered extremely significant (^***^) change compared with control.

The anti-cancer efficacy of aPPD was elucidated using nude mice bearing human C4-2 prostate tumor xenografts developed following subcutaneous injection of C4-2 human prostate cancer cells. The control group received only the vehicle formulation (ethanol: propylene glycol: water in 2:7:1 v/v/v ratio). During this study, aPPD produced significant inhibition of the C4-2 tumor growth rate starting on day 7 and onwards for up to 46 days compared to the control group (p <0.05) (Figure 2A). The maximum inhibition of tumor growth was seen after 7 days of treatment and a sustained tumor suppressive effect was observed until 46 days of aPPD treatment (euthanasia point) with 53% inhibition compared to the control group (Figure 2A).

The average tumor volume for control treated animals was approximately 6-7 times the size of the average tumor volume determined when treatment was initiated. For animals treated with aPPD, the tumor volumes were 3 to 4 times greater than the treatment initiation time point and the tumors at this time were significantly smaller than those tumors from animals treated with the formulation vehicle. In addition, aPPD had significantly different serum PSA levels after 7 and 14 days (Figure 2B). PSA levels decreased at week 1, 2 and week 6 with ~46% and 34% inhibition, respectively, compared to control, followed by no difference with the control group between week 2 and week 5. Interestingly, the PSA levels demonstrated a significant 27% decrease again at week 6 following treatment initiation.

### Lack of toxicity from aPPD treatment

There was no difference in animal body weight between vehicle- and aPPD-treated mice during the study period, indicating that the selected dose is safe and well tolerated (Figure 2C), which is in agreement with our previous observations in other PCa xenografts models [12, 18, 19]. Histopathological evaluations of the lung, liver, kidney and spleen from control or aPPD-treated mice show no signs of abnormal findings (Supplementary Figure 1). In addition, liver and kidney function tests do not reveal any organ toxicity following treatment (Supplementary Table 1). Interestingly, serum amylase (AMY) levels following aPPD administration were significantly lower than the control group. We therefore measured the levels of lipase enzyme, LIP, another pancreatitis marker, for which there were no significant differences between the two groups. Overall, both AMY and LIP levels were within the normal reported range in the literature [22–25]. There was no statistical difference in serum albumin (Alb), alkaline phosphatase (ALP), alanine aminotransferase (ALT) and alanine transaminase (ALT) values which were within the expected range for normal mice. Serum creatinine levels were determined as a measure of kidney toxicity and the results suggest that there was no significant difference in serum creatinine levels between the aPPD and the control (Supplementary Table 1). Overall, there were no significant differences in the histological findings between the control and aPPD treatment group in any of the tissues examined (liver, lungs, kidneys, and spleen). Therefore, indicating that aPPD treatment was safe at the therapeutic doses used in the present study.

### aPPD inhibits proliferation and induces apoptosis in C4-2 xenograft tumors

**Figure 3:**
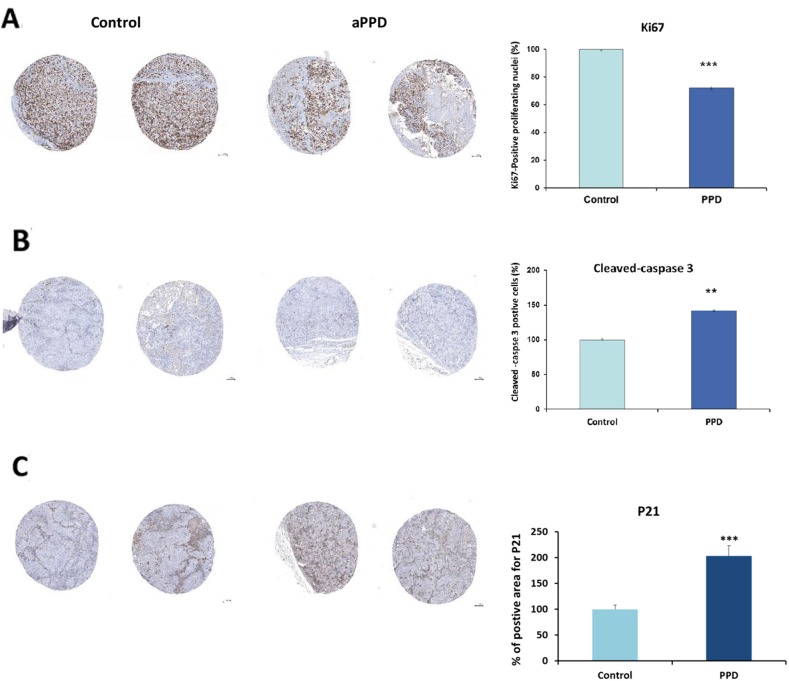
IHC staining of tumors derived from C4-2 xenografts **(A)** Effects of aPPD on C4-2 tumor cell proliferation. **(B)** Effects of aPPD on apoptosis marker cleaved caspase-3 in the tumors. **(C)** Effects of aPPD on cell cycle inhibitor p21 in the tumors. C4-2 cell xenograft tumors were excised after 46 days of treatments with aPPD or control. Data are presented as Mean ± SEM, n of 4. A *p* value < 0.05 was considered significant (^*^), A *p* value < 0.01 was considered very significant (^**^) and a *p* value < 0.001 was considered extremely significant (^***^) change compared with control.

The C4-2 tumors were harvested from mice following 46 days of treatment (once daily) and were subjected to immunohistochemical analyses for proliferation (Ki-67 labeling), cell cycle regulator (p21), and apoptosis (Bax and cleaved caspase-3). As shown in Figure 3A, aPPD significantly inhibited cell proliferation as measured by Ki-67 labeling and the extent of suppression was approximately 25% lower than that observed in tumors isolated from animals treated with vehicle alone. Tumors from mice treated with aPPD experienced a 40% increase (p<0.001) in the number of apoptotic cells compared to control mice (Figure 3B). Bax is a pro-apoptotic protein, which inhibits caspase-3 activity, and therefore attenuates apoptosis. In the present study, consistent with previous *in vitro* results, an increase in Bax and cleaved caspase 3 levels were detected in the C4-2 tumors treated with aPPD. This was confirmed upon immunostaining for cleaved caspase-3 in the tumor sections from aPPD-treated and vehicle-treated groups. Thus aPPD anticancer activity appears to be mediated through mechanisms that cause a decrease in cell proliferation as well as an increase in apoptosis.

### aPPD downregulates AR protein levels in C4-2 xenograft tumors

**Figure 4:**
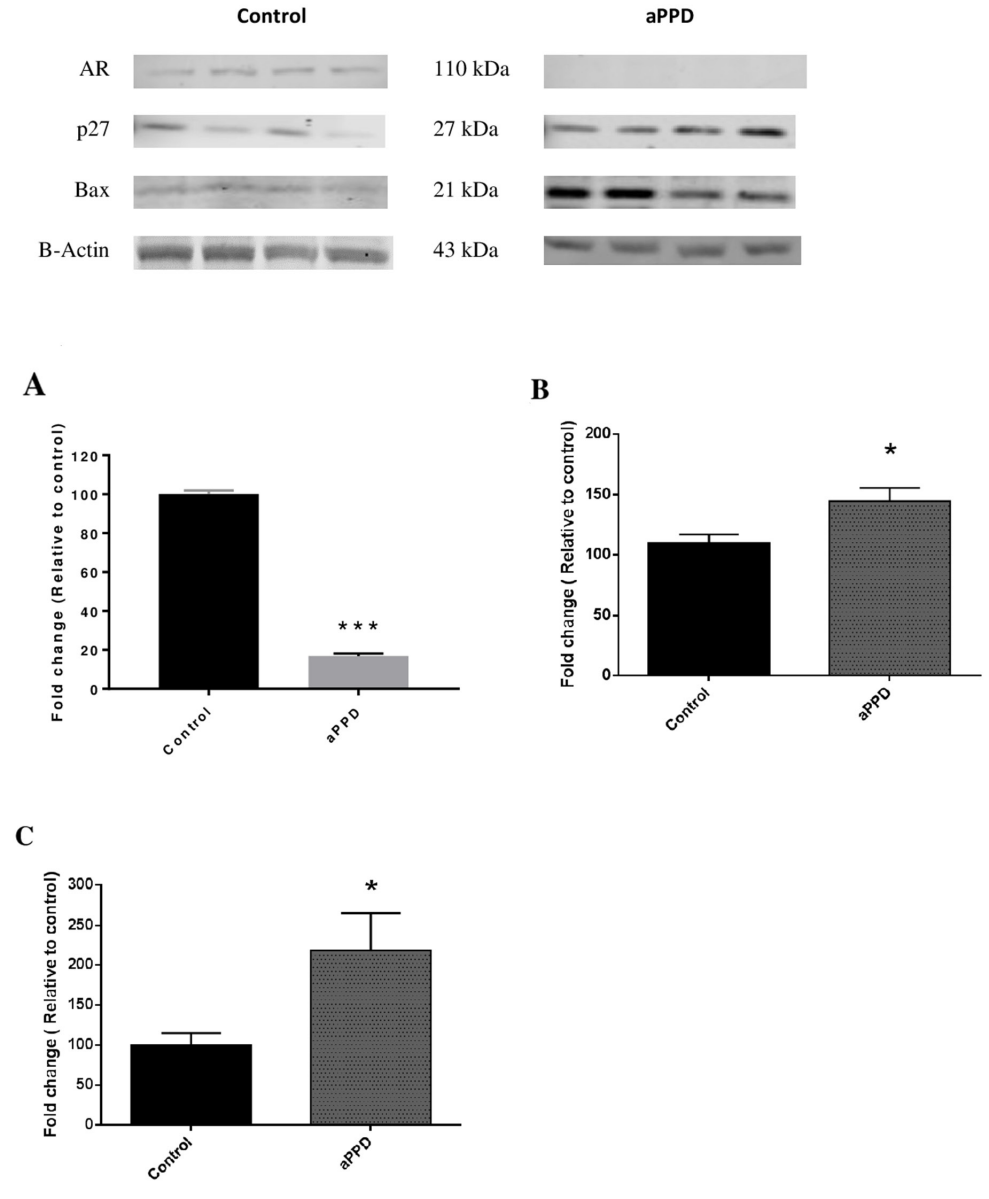
Representative Immunoblots and quantitative analyses of protein levels in C4-2 xenograft tumor as determined by Western blot AR protein **(A)** is downregulated in four aPPD-treated tumors. p27 and Bax proteins **(B and C)** are upregulated in four aPPD-treated tumors.). The experiments were performed in duplicate and expressed as Mean ±SEM. A *p* value < 0.05 was considered significant (^*^), A *p* value < 0.01 was considered very significant (^**^) and a p value < 0.001 was considered extremely significant (^***^) change compared with control (vehicle-treated group).

To elucidate the mechanistic aspect of aPPD-mediated C4-2 tumor suppression, AR protein levels were determined using Western Blot analyses. Strong downregulation of AR protein expression was seen in aPPD treated C4-2 xenograft tumors. Relative quantification of AR protein to beta actin shows that aPPD blocks AR expression by 84% compared to the control mice in C4-2 tumors (Figure 4).

### aPPD causes cell cycle arrest

A significant upregulation of p27 and p21 protein was observed in tumors derived from aPPD treated mice. Results show that aPPD induces cyclin dependent kinase inhibitors (CKI) p27 and p21, leading to decreased cyclin dependent kinase activity and cell cycle arrest in G1 phase. An increase in p21 expression and enhanced p27 accumulation correlates with the sensitivity of C4-2 tumors to aPPD treatments as shown in Figure 2.

### *In silico* analysis of aPPD binding to AR ABS

**Figure 5:**
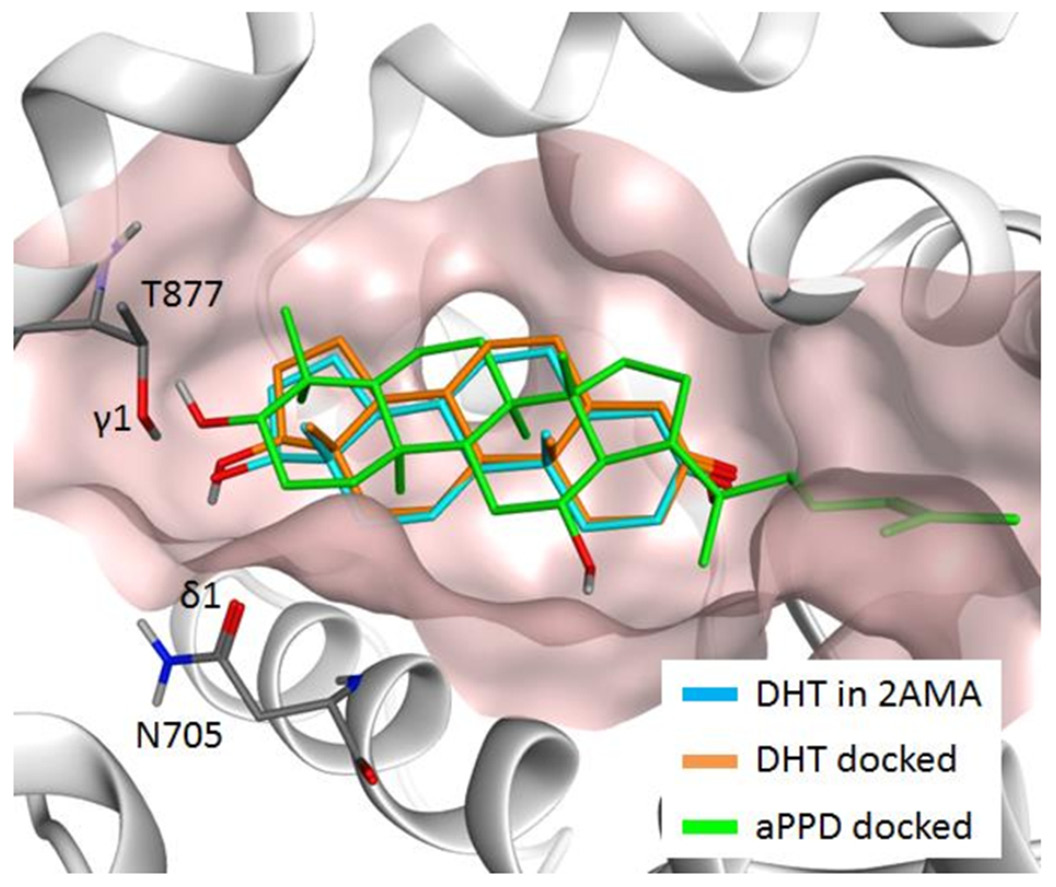
Predicted docking poses of dihydrotestosterone (DHT) (orange) and aPPD (green) in AR ABS, along with the DHT (light blue) in the 2AMA X-ray structure

Figure 5 presents the predicted docking poses of DHT (orange) and aPPD (green) in the AR ABS, along with DHT (light blue) in the 2AMA X-ray structure. It was found that the docking pose of DHT of the 2AMA X-ray structure and the docking pose of DHT predicted by Vina are almost identical (the root mean square deviation between them is 0.26 Å), demonstrating the Vina's capability to predict the correct binding pose. It was also determined that aPPD can be docked into ABS without serious steric hindrance, and the four rings of aPPD occupy the similar space that is occupied by the four rings of DHT, but with a slightly different orientation. The docking score of DHT was calculated to be −11.1, while that of aPPD was predicted to be −7.1. A closer look at the binding orientations, delineate that the 17β hydroxyl group of DHT forms hydrogen bonds with both ASN705 and THR877, as has been observed in a previous X-ray study [26]. The distance between the oxygen atom (O_17_) of the 17β hydroxyl group of DHT and the oxygen δ1 of ASN705 is 2.7 Å, and the distance between O17 and the oxygen γ1 of THR877 is 2.8 Å. On the other hand, aPPD can form the hydrogen bond with THR877, but with ASN705. The distance between the oxygen atom of the hydroxyl group of aPPD and the oxygen γ1 of THR877 is 2.7 Å, but the distance between the oxygen atom of aPPD and the oxygen δ1 of ASN705 is now 3.9 Å. The elongation of the oxygen-oxygen distance between aPPD and ASN705 occurred mainly due to the existence of two methyl groups attached to the carbon atom adjacent to the hydroxyl group (DHT has only one methyl group attached to the corresponding carbon atom), and also the fact that aPPD needs to accommodate its methylheptyl tail into the ABS. Considering that two hydrogen bonds that 17β hydroxyl group forms with ASN705 and THR877 are conserved among the testosterone, DHT, and tetrahydrogestrinone [26], and also R1881 [27], both hydrogen bonds can be the key interactions that ABS needs to accommodate its ligands. We suggest that aPPD is a weaker binder for ABS because aPPD does not form one of the two key hydrogen bonds.

### aPPD suppresses AR transactivation

**Figure 6:**
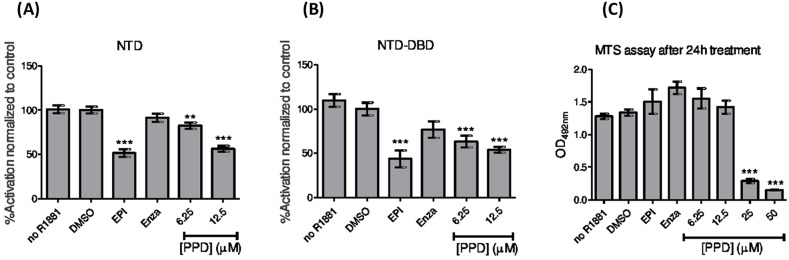
Inhibition of the androgen receptor by aPPD Inhibition of the isolated N-terminus domain (NTD) **(A)** and combined NTD and the DNA binding domain (NTD-DBD) **(B)** of AR by aPPD. **(C)** The toxicity of aPPD was assessed in the same experimental conditions on non-transfected PC3 cells using an MTS cell viability assay. The enzalutamide (Enza, C-terminus inhibitor) and EPI-001 (EPI, N-terminus inhibitor), and non-stimulated (no R1881) were used as controls. The results represent the mean ± SEM of 3 independent experiments with 6 replicates each.

We have shown that aPPD (70 mg/kg daily 5 times every week for 4 weeks) was highly effective in inhibiting PC-3 tumor growth *in vivo* [19]. In this study, the toxicity and effect of aPPD on AR activity was assessed in non-transfected PC-3 cells treated with increasing concentrations of this inhibitor, using a cell viability MTS assay. Up to a concentration of 12.5 μM of PPD, there was no effect on the cell viability of PC-3 lacking the androgen receptor (Figure 6C). However, aPPD at 25 μM and 50 μM demonstrated significant cellular toxicity. PC-3 cells lacking the AR activity were co-transfected with either NTD or combined NTD-DBD, followed by treatment with an ABS inhibitor (enzalutamide) or an N-terminus inhibitor (EPI-001). Except for the control cells, all the treatment groups were treated with R1881, a synthetic androgen and potent AR activator. Similar to the N-terminus inhibitor EPI-001, [28], aPPD (6.25 μM and 12.5 μM) was able to significantly inhibit both NTD- and NTD-DBD-mediated AR activation while the C-terminus inhibitor enzalutamide did not affect the activity (Figure 6A–6B).

## DISCUSSION

The effective treatment of CRPC remains a challenge. It is well established that the role of the AR persists following androgen deprivation therapy and that this very well defined therapeutic target acquires resistance via multiple evasive mechanisms [29]. In such a dynamic progressive disease, it is essential that we come up with targeted strategies that are pleiotropic by nature in order to thwart rapid onset of advanced stages of prostate cancer. In numerous models for prostate cancer, we have identified and reported on multiple mechanisms of action of aPPD, a naturally derived compound found in ginseng [2, 12, 18, 19]. Based on our recent findings, that aPPD may have superior anticancer activities in C4-2 cells than LNCaP PCa cells *in vitro*, we carried out an *in vivo* study to examine the aPPD influence on AR-positive human C4-2 prostate xenograft tumors in mouse [2]. Furthermore, to better understand the molecular mechanisms of aPPD-mediated anticancer effects, we explored the potential binding of aPPD to multiple sites on the AR protein *in silico* to further rationalize its effect on AR binding and activation *in vitro*.

We demonstrated that the ginsenoside aPPD significantly suppresses C4-2 tumor growth in mice bearing prostate cancer xenografts following treatment with oral gavage for 46 days. The inhibition of tumor growth was evident after seven days of treatment and the effect was pronounced as the treatment period increased. Similarly, PSA levels also decreased following aPPD treatment. The treatment dose was selected based on a previous study in PC-3 cells [12, 19]. Treatment with aPPD did not cause any acute toxicity in the xenograft model as determined by bodyweight, physical appearance, behavior or food and water intake. In keeping with this, upon harvesting of blood and organs, the liver and kidney function tests performed after aPPD treatment did not show any statistically significant change as indicated by ALT, AST and ALP, and serum creatinine levels, suggesting no organ-based toxicity. Although we observed a decrease in serum amylase in aPPD-treated mice compared to the control group, the levels were still in the normal range. The accompanying markers of chronic pancreatitis were absent which therefore suggests no pancreatic abnormality. In chronic pancreatitis, amylase (AMY) and lipase (LIP) may be normal or decreased and lipase production can drop to less than 10% of the normal level [30]. The AMY and LIP levels were within the normal range reported in the literature [22–25] and there were no significant differences in LIP levels between the two groups. In addition, glucose levels were within the normal range in both groups. Collectively, this data indicates that aPPD treatment was safe at the therapeutic doses. This is consistent with our previous work (12) where a ternary solvent system containing ethanol, propylene glycol and water (2:7:1) was used to formulate the aPPD for oral gavage, as per previous published work [12]. Our previous studies have established that the ternary solvent mixture is not toxic by itself when used in limited volume [12, 18, 19]. This is the first report of aPPD-mediated antitumor activity in C4-2 prostate cancer model representing castration resistant disease. In corroboration with the data presented here, Cao et al. [16, 17] have also shown previously that aPPD inhibits the growth of LNCaP xenograft tumors (androgen-dependent) and castration-resistant 22Rv1 xenograft tumors.

To determine the mechanism of anti-tumor activity of aPPD, the C4-2 tumors were excised after the treatment period and markers of apoptosis (Bax and cleaved caspase-3) and proliferation (Ki-67) were measured. Initiation and progression of PCa are characterized by alterations and disruption in the regulatory pathways of AR, apoptosis and cell cycle regulation. Ki-67 is a marker of proliferation and can assist in the predictions of prostate cancer outcome (survival and prostate cancer recurrence) [31–34]. The results from the current study confirm our previous finding that aPPD is an inhibitor of the Ki-67 proliferation marker and a stimulator of caspase-3 function that can induce apoptosis in PCa *in vivo* (12). As measured by Ki-67 labeling, aPPD significantly inhibited cell proliferation and the extent of suppression was significantly lower than what was observed in tumors isolated from mice treated with vehicle alone (Figure 3A). We have previously shown that aPPD lowers cell proliferation in PC-3 androgen-independent prostate tumors *in vivo*. In addition, aPPD is a strong promoter of apoptosis in C4-2 androgen-dependent prostate cancer cells as well as in LNCaP androgen dependent cells *in vitro*, and in PC-3 androgen-independent prostate cancer xenografts *in vivo* [2, 12, 18, 19]. Bax is a pro-apoptotic protein, which can inhibit caspase-3 activity, and contribute to reduced apoptosis. In the present study, consistent with previous *in vitro* results, an increase in Bax expression was detected in the C4-2 tumors treated with aPPD. Thus, aPPD-induced apoptosis may be associated with activation of the Bax/caspase-3 pathway. It has been reported that aPPD significantly upregulates Bax protein expression in LNCaP and C4-2 cells increasing the expression of cleaved caspase 3 in the C4-2 cell line *in vitro* [2]. Other studies have also shown that ginsenosides are significant inducers of apoptosis and inhibit proliferation in prostate cancer models *in vitro* [14, 35, 36]. These data are consistent with those shown in Figure 3B, where aPPD caused a significant increase in apoptotic index relative to tumors from control animals. We conclude therefore that aPPD has multiple anticancer activities which have both anti-proliferative and pro-apoptotic mechanisms.

It is well understood that AR protein is a central driving force in prostate cancer that persist in CRPC. C4-2 is an AR-dependent cell line, the effects of aPPD on AR protein expression and activity were thoroughly examined as part of this study. In spite of the close resemblance of aPPD to testosterone/DHT, aPPD is not likely a competitive antagonist of AR [16, 17]. Rather, aPPD was found to influence AR protein expression levels and consequent functionalities [16, 17]. Quantification of AR protein levels in C4-2 xenograft tumors suggests that aPPD has the ability to downregulate AR expression and decrease in the PSA serum levels. Differences in PSA serum levels were significant for the first 2 weeks and during the last week only in aPPD treated samples compared to control. Serum PSA does not predict tumor volume but is dependent to a significant degree on the growth rate of the tumor. A rapidly growing tumor does not always predictably lead to an increased PSA level. In addition, a decrease in PSA does not necessarily correlate with increased cell death. A wide range of rates of tumor cell death are exhibited by different types of tumors and depend on the specific agent, its concentration and the type of cell lines evaluated [37]. It has been reported that correlations between PSA and tumor volume decrease over time, ultimately PSA correlates with prostate size but not necessarily with tumor volume [37]. In our study, the Pearson's correlation of mean PSA with mean tumor volume in aPPD treated group was R^2^ =0.4471 (Supplementary Figure 2). By contrast, PSA in the control group was more robust and had a stronger correlation with tumor volume as indicated by a Pearson value of 0.8226, p<0.001. In accordance with this finding, Cao et al. [16, 17] have also shown that aPPD downregulated AR expression in LNCaP xenograft tumors and it is suggested that multiple mechanisms may be involved in the aPPD-mediated downregulation of AR expression. Induction of proteasome-mediated degradation of AR protein was the primary mechanism of AR regulation within the initial 12 hr of aPPD treatment in studies conducted by Cao et al. [16, 17]. It is postulated that blockade of interaction of N-terminus and C-terminus of AR protein instigates the AR degradation cascade. However, aPPD was also shown to subsequently decrease the promoter activities by 80% leading to decreased AR transcription [16]. The *in vivo* results in the current study are consistent with our previous *in vitro* observations [2] as well as other studies reported in a variety of prostate cancer cell lines including LNCaP and 22RV1 prostate cancer cells [2, 9, 13, 16, 17, 38].

It has been reported that aPPD downregulates the transcription of full length and AR variants lacking the LBD and that the suppression of the AR transcriptional activity is not affected by increasing concentrations of androgen [16, 17]. These data suggest that aPPD binds to either the NTD or the DBD domain of AR. In order to evaluate this hypothesis, we transfected AR negative PC-3 cells with either the isolated NTD or the combined NTD-DBD domains and evaluated the effect of aPPD on these constructs. To explore the potential direct binding site of aPPD on AR, we performed *in silico* docking simulation by targeting three functional binding sites, the ABS, the activation function 2 site (AF2), and the binding function 3 site (BF3). Our docking results suggest that aPPD weakly binds to the ABS compared to DHT, because aPPD does not form one of the two key hydrogen bonds which is consistent with the observation made in the previous study [16]. This is the first report of aPPD docking to the different functional domains of AR.

Typically, binding of a molecule to AR may have growth stimulatory or suppressive effects. So, to evaluate the implications of aPPD binding with AR protein, we elucidated the effect of aPPD on transactivation of AR, either through NTD or combined NTD-DBD components. Interestingly, aPPD at 6.25 μM or 12.5 μM concentration inhibited AR activation in the presence of the potent AR agonist, R1881. These results suggest that the interaction of aPPD with AR occurs at multiple binding sites, leads to inhibition of AR activation and ultimately AR-mediated tumor growth suppression. The aPPD-mediated AR suppression observed in this study is likely to influence the CRPCs that are functional AR-dependent. Additionally, inherent multiple anticancer mechanisms of aPPD [3, 9, 16, 17, 39–42] facilitate effective inhibition of both C4-2 androgen-dependent xenograft growth and androgen-independent PC-3 cells [2, 12]. It is worth noting that in the present study aPPD outperforms enzalutamide in that its activity goes beyond LBD binding to AR and it inhibits NTD or combined NTD-DBD transactivation. This corroborates the work of Cao and Rennie et al. (2013) who previously demonstrated that AR and its splice variants may be inhibited by aPPD [16].

**Figure 7:**
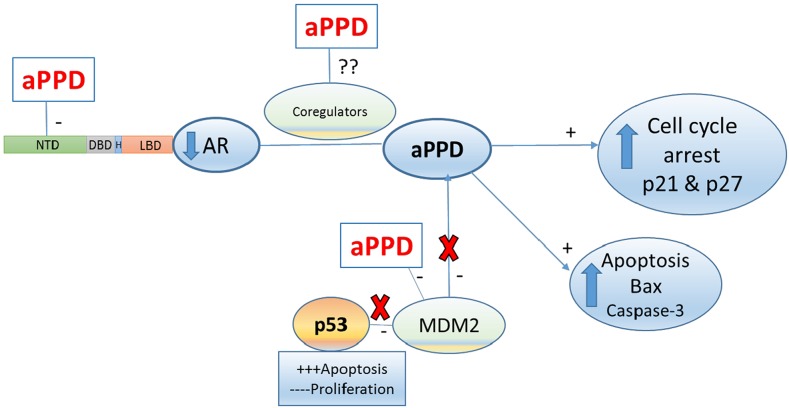
Proposed aPPD anti-prostate cancer mechanism in C4-2 model of castration-resistant prostate cancer aPPD inhibits AR signaling pathway via inhibition of a site in the AR N-terminal domain. aPPD upregualted p21, p27, Bax and cleaved Caspase 3 levels in C4-2 tumors. The AR downregulation appears to be secondary to suppression of MDM2 in aPPD treated mice. aPPD binds to MDM2 proteins and activate p53 apoptosis signaling pathway, thus induce apoptosis and cell cycle arrest. This may result from indirect inhibition of the MDM2 by aPPD metabolite. Probably these levels of aPPD metabolites may be sufficient to promote p53 activation as well. An amino-terminal domain (NTD), DNA-binding domain (DBD), hinge region (H), ligand-binding domain (LBD). Mouse double minute (MDM2).

Consistent with previously reported studies [2, 14, 38, 43], aPPD treatment leads to increased levels of p21 and p27 and an accumulation of cells in G1 phase of the cell cycle. A decrease in p21 expression and enhanced accumulation of p27 correlates with the observed sensitivity of C4-2 tumors to aPPD treatments as shown in Figure 2. A recent study has also shown that a aPPD metabolite (25-OH-PPD) significantly induced apoptosis by upregulating Bax, causing an increase in cleaved caspase-3 via binding and downregulation of MDM2 oncoprotein in PC-3 xenograft tumors (AR negative) [15]. MDM2 is a potent negative regulator of p53 that works via enhancement of P53 protein degradation [44]. MDM2 also has p53-independent functions in cellular differentiation processes and signaling and is known to interact with AR protein [45]. Tovar et al. [46] have shown that MDM2 antagonist (nutlin-3a) in combination with androgen depletion *in vitro* and *in vivo* additively increased apoptosis and further downregulated AR expression in AR positive LNCaP (androgen-dependent) and 22Rv1 (androgen-independent) cell lines. This was secondary to p53 activation [46]. MDM2 antagonism also led to a greater tumor regression and dramatically increased survival in LNCaP-bearing nude mice (p53 wild type PCa) [46]. Here, we examined the effect of aPPD in C4-2 xenograft tumors which are androgen independent - albeit AR positive. We therefore speculate that AR downregulation was secondary to knockdown of MDM2 in aPPD treated mice. Overall, aPPD led to elevated levels of p27 and p21, and enhanced cell cycle arrest and apoptosis through p53-dependent and -independent mechanisms (Figure 7).

In summary, the ginseng derived ginsenoside aPPD inhibited C4-2 tumor growth by 53% compared to control treatment and, in accordance with this, serum PSA was decreased by 25%. Further, the IHC and Western blot analysis of excised tumors showed that tumor cell proliferation rate (measured by Ki-67 positive cells) was significantly lower for aPPD, and that was associated with elevated levels of Bax and cleaved caspase-3 expression (apoptotic markers), compared to the mice treated with vehicle alone. In addition, aPPD led to a significant increase in p21 and p27 (cell cycle inhibitors) protein levels. Furthermore, our finding that aPPD downregulated AR expression *in vivo* taken in combination with *in silico* and *in vitro* studies suggest that aPPD binds and significantly inhibits the NTD or the DBD domain of AR. The novel findings described by this study include aPPD potently inhibits PCa *in vivo* via inhibition of a site on AR N-terminal domain and concurrently induces apoptosis. These preclinical results support testing of aPPD in a clinical setting in advanced human PCa patients. Further research will be needed to determine whether aPPD treatment can target AR for the treatment of CRPC patients.

## MATERIALS AND METHODS

### Test compound and reagents

Ginsenoside aPPD (MW 460.73 g/mol, with a purity of ~98.9%, which was confirmed in our lab by using LC-MS), was provided as a gift by the Shanghai Innovative Research Center of Traditional Chinese Medicine (Shanghai, China). High-performance liquid chromatography grade chemicals and all other chemicals were obtained from Sigma–Aldrich Canada Ltd. (Oakville, ON, Canada) and Fisher Scientific (Ottawa, ON, Canada).

### *In vivo* studies

#### Xenograft preparation and treatment

All animal experiments were conducted in accordance with the University of British Columbia's Committee on Animal Care and protocol # A11-0377 held by Dr. Guns at the Vancouver Prostate Centre. Male athymic mice age 6–8 week old (Harlan Sprague Dawley, Inc.) weighing 25–31 g were used in our study. Two million C4-2 cells in 0.5 mL (Matrige, BD Biosciences), were subcutaneously inoculated at the posterior dorsal site, similar to previous experiments (14). When serum PSA levels reached more than 25 ng/ml, mice were castrated. Post-castration, animals were monitored and when PSA recovered to pre-castration levels, 20 mice were randomized and distributed into two treatment groups: Treatments began once the total tumor size exceeded 100 mm^3^ with either aPPD at 70 mg/kg once daily (117–150 μl) or the vehicle control at an equivalent volume based on weight, with a total of 8 mice per group.

#### Oral gavage formulation

The ginsenoside aPPD was formulated just prior to oral administration as previously described by our laboratory [12]. Briefly, aPPD solubilized in ethanol: propylene glycol: water (2:7:1, v/v/v ratio) was prepared prior to the administration by oral gavage at a dose of 70mg/ kg (highest achievable dose, limited due to gavage volume limitations (150 μl) implemented by the institutional animal care committee). Dose selection was based on previous work completed with aPPD in our lab for safety, solubility and potency determined in solvents amenable to animal dosing prior to optimizing formulation for animal studies.

#### Assessment of tumor growth and PSA

Tumor size (mm3) was measured and monitored twice weekly. Calipers (volume ¼ length width weight 0.5326) were used to measure the three perpendicular axes of each tumor to calculate the tumor volume. PSA levels were measured by tail vein sera samples weekly using the Cobas automated enzyme immunoassay (Montreal, PQ, Canada).

#### Assessment of toxicity

During treatment, aPPD toxicity was determined. Animals were monitored daily for changes in body weight (g), appearance and signs of acute toxicity including death, lethargy, blindness, and disorientation. Mice were sacrificed when tumor volume exceeded 1,500 mm^3^ or loss of > 20% body weight. All xenograft tumors were harvested after 46 days of the treatment approximately 24 hours after their last treatment dose. Blood samples were collected for CBC, liver and kidney function tests, serum electrolytes, glucose, serum albumin and total blood protein levels. In addition, liver, spleen, kidney, lung and brain tissues were collected for further toxicological and histopathological analysis.

#### Tumor collection and homogenization

At the end of the treatment period (46 days after treatments) mice were sacrificed and tumors were harvested and divided into two fractions: either frozen in liquid nitrogen and stored at −80 °C for protein analysis or preserved in 10% formalin buffer and tissue sections embedded in paraffin blocks for histopathological analysis. Preparation of paraffin-embedded tissue sections and immunohistochemical analyses were carried out as previously described [47, 48].

#### Western blot analysis

Excised C4-2 tumor tissue was homogenized using the Precellys™ tissue homogenizer system (Bertin Technologies, France) as per the manufacturer's protocol. Proteins were extracted using RIPA buffer and Western blot was performed as previously described. Briefly, tumor tissue (100 mg) was homogenized in RIPA buffer with 1X protease inhibitor at a 1:4 (tissue: buffer) ratio using Precellys™ Tissue Homogenizing CKMix (Cat. # 3961-1-009) at 6000 rpm for two cycles of 20 s each with a 15 s break. Thirty micrograms of protein were loaded per lane into 12% SDS-acrylamide gels. After electrophoresis, proteins were transferred to nitrocellulose membrane in 48 mM Tris, 39 mM glycine, 0.1% SDS and 20% methanol (pH 8.3). The membranes were then blocked using Odyssey blocking buffer (Li-COR) containing 5% non-fat milk in wash buffer (Dulbecco's phosphate-buffered saline with 0.1% Tween 20) for 2 h and incubated overnight at 4 °C with primary antibodies, followed by at room temperature for 3 h. Subsequently, membranes were washed and incubated in Odyssey secondary antibody for 30–45 min according to manufacturer's instructions. Blots were imaged using an Odyssey Infrared Imaging System (LI-COR Biosciences, Lincoln, NE, USA). Quantification was performed on single channels with the analysis software provided and normalized to beta actin for loading and transfer. The fold induction or reduction of AR proteins was compared to that of the vehicle control group. Antibody dilutions, duration of second antibody incubation and film exposure were optimized to produce bands linearly related to the amount of protein. The following antibodies and dilutions were used to develop the immunoblots: mouse monoclonal antibody for beta actin as loading control (1:5000; Sigma–Aldrich), rabbit polyclonal anti-p27 (1:250; Santa Cruz Biotechnology Inc.), mouse monoclonal antibody for AR (1:200; Santa Cruz Biotechnology Inc.), and rabbit monoclonal anti-Bax (1:1000;Abcam). Conjugated secondary antibodies (anti-mouse IRDye 800 at a dilution of 1:5000 and anti-rabbit IRDye 680 at a dilution of 1:20,000) were obtained from Cedarlane Laboratories (Burlington, ON, Canada).

#### Immunohistochemistry

C4-2 tumors were isolated from mice at the end of the study described above and were prepared for immunohistochemical assessment of apoptosis and Ki-67. C4-2 tumors were sectioned and stained with hematoxylin and eosin (HE) and the desired areas marked along with their corresponding paraffin blocks. The rabbit anti-human anti-Cleaved Caspase 3 (Asp175) (5A1E) (1:50; Cell Signaling Technology, Danvers, MA, USA), rabbit anti-human anti-Ki 67 proliferating markers (1:50; Cell Signaling Technology, Danvers, MA) and rabbit polyclonal anti-p21 (1:150; Santa Cruz Biotechnology Inc) were used for immunohistochemical staining. All sections used for immunohistochemistry were lightly counterstained with 5% (w/v) Harris hematoxylin. Five fields of each slide were randomly chosen and images taken (400), using an AxioCam HR CCD mounted on an Axioplan 2 microscope and Axiovision 3.1 software (Carl Zeiss, Canada). Positively stained cells and whole cells in each image were counted and the percentage of positive cells was calculated. The TMAs were manually constructed (Beecher Instruments, MD, USA) by punching quadruplicate cores of 1 mm for each sample giving a total of 144 cores. All scoring was done blinded with respect to treatment by LF and based on relative immunoreactive intensity on a four-point scale.

### *In silico* docking between aPPD and AR ABS

To gain insight of into why aPPD cannot compete with DHT for ABS in spite of its structural similarity, we performed *in silico* docking study for DHT and aPPD against AR ABS to seek the possible molecular mechanism. The X-ray crystal structure of AR LBD complexed with DHT was obtained from the Protein Data Bank (PDB ID: 2AMA), and AutoDock Vina [C2] was employed for the *in silico* docking [49]. The protein model was prepared with Molecular Operating Environment (MOE) 2015.1001 [C3] by adding the missing residues and the side chains to the protein coordinate in the X-ray structure [50]. The center of the binding pocket was defined based on the coordinate of DHT ligand in the X-ray structure, and the box dimension of 24 Å × 24 Å × 24 Å was used for the grid search which is large enough to accommodate the ligand molecule.

### *In vitro* aPPD-AR binding and inhibition assays

PC-3 cells lacking the AR and authenticated by IDEXX Laboratories (Maine, USA) were maintained in RPMI 1640 media (Life Technologies) and 5% FBS (Hyclone Thermo Fisher Scientific) at 37 °C and 5% CO2. Cultures were routinely monitored for mycoplasma contamination. Cells were seeded in 96-well plates (5,000 cells/well) in RPMI 1640 medium with 5% charcoal-stripped serum (CSS) (Hyclone). After 24 h, cells were co-transfected with both NTD and pG5luc (10 ng each) or NTD-DBD and pARR3-tk-luciferase (5 ng each) using TransIT20/20 transfection reagent (3 μL/μg of DNA) (Mirus Bio LLC, Madison, WI, USA) in Optimem serum-free media (Life Technologies) for 24 h according to the manufacturer's suggested protocol. At 24 h after transfection, cells were treated with either 0.1% DMSO (solvent control) or serial dilutions of increasing concentrations of aPPD. Enzalutamide (C-terminus inhibitors) and EPI-001 (N-terminus inhibitor), were used as positive controls. At 24 h after treatment, the medium was aspirated off and the cells were lysed by adding 60 μL of 1× passive lysis buffer (Promega) followed by shaking at room temperature for 15 min and two freeze/thaw cycles at −80 °C. Twenty microliters of lysate from each well were transferred onto a 96-well white flat bottom plate (Corning) and the luminescence signal was measured after adding 50 μL of luciferase assay reagent (Promega). The chemical oxidation of luciferin into oxyluciferin by the luciferase is accompanied by light production that can be quantified as luminescence by a TECAN M200Pro instrument. Each concentration was assayed in replicates n = 6, with a biological replicate of n = 3. The toxicity of aPPD (6.25-50 μM) was assessed in the same experimental conditions on non-transfected PC-3 cells using the 3-(4,5-dimethylthiazol-2-yl)-5-(3-carboxymethoxyphenyl)-2-(4-sulphophenyl)-2*H*-tetrazolium (MTS) assay.

### Statistical analysis

For each studied variable, mean and standard error of the mean (SEM) were calculated. Differences between the mean values of two treatment groups were analyzed using the Student *t*-test (parametric). The level of significance was set prior at a *P* value of < 0.05.

## SUPPLEMENTARY MATERIALS FIGURES AND TABLES


