# Supplementary material for: 20(S)-protopanaxadiol regio-selectively targets androgen receptor: anticancer effects in castration-resistant prostate tumors
Source: Oncotarget. 2018 Apr 20;9(30):20965–78. doi: 10.18632/oncotarget.24695 (PMC5940378; doi:10.18632/oncotarget.24695)
Supplement: Supplementary file 1 [file oncotarget-09-20965-s001.pdf]

20(S)-protopanaxadiol regio-selectively targets androgen receptor: anticancer effects in castration-resistant prostate tumors

SUPPLEMENTARY MATERIALS

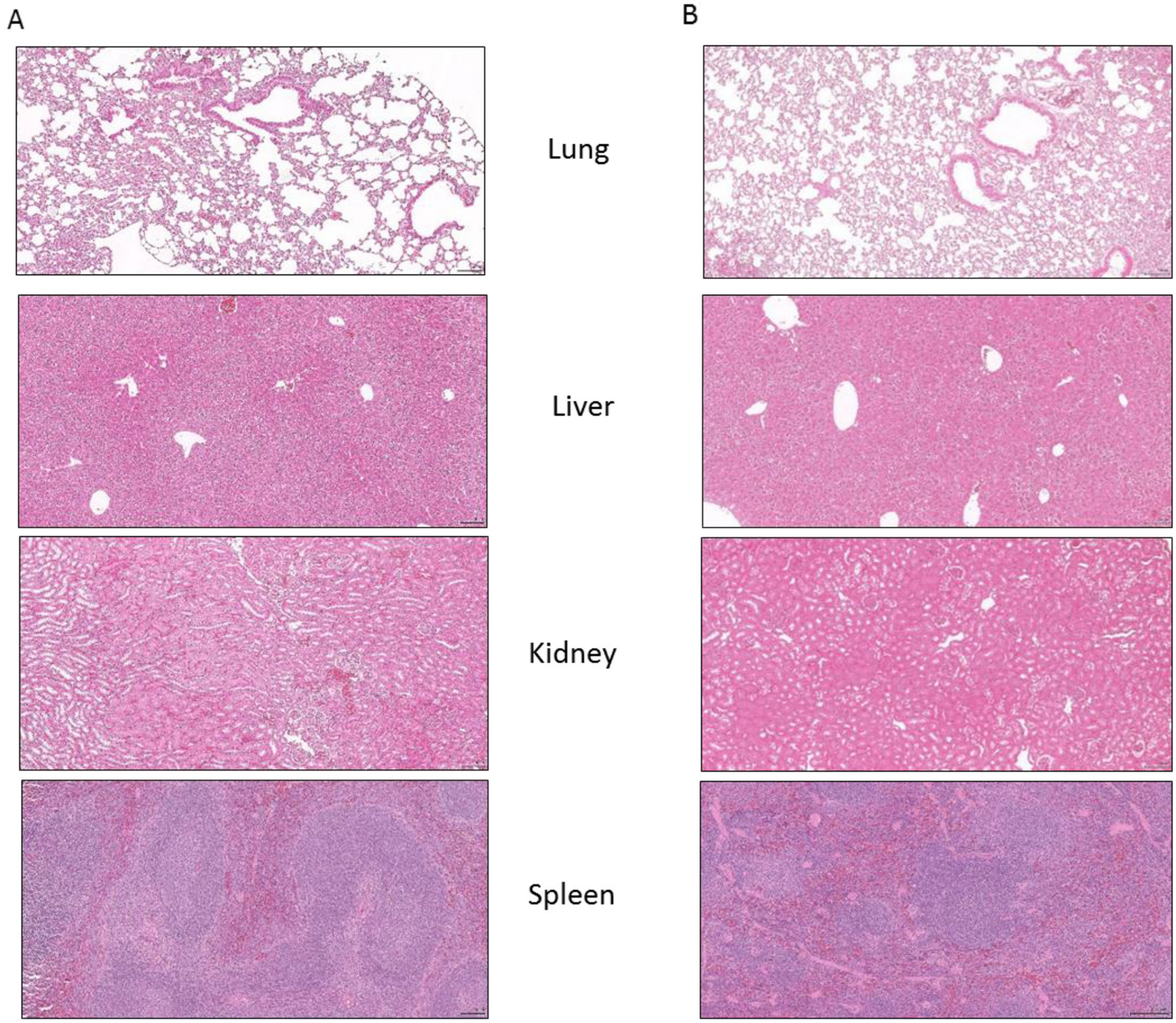

Supplementary Figure 1: Histopathological evaluation of the organs of male nude mice treated with aPPD for 6 weeks. Histological analysis of the organs (the lungs, liver, kidneys, and spleen) from the control group **(A)** and from the mice treated with aPPD (70 mg/kg) **(B)**. No abnormal histopathological findings were observed. Samples were stained with hematoxylin and eosin (H&E. Digital image were obtained by using the Leica SCN400 scanning system with the SL801 autoloader (Leica Microsystems; Concord, Ontario, Canada) at magnification equivalent to 10x.

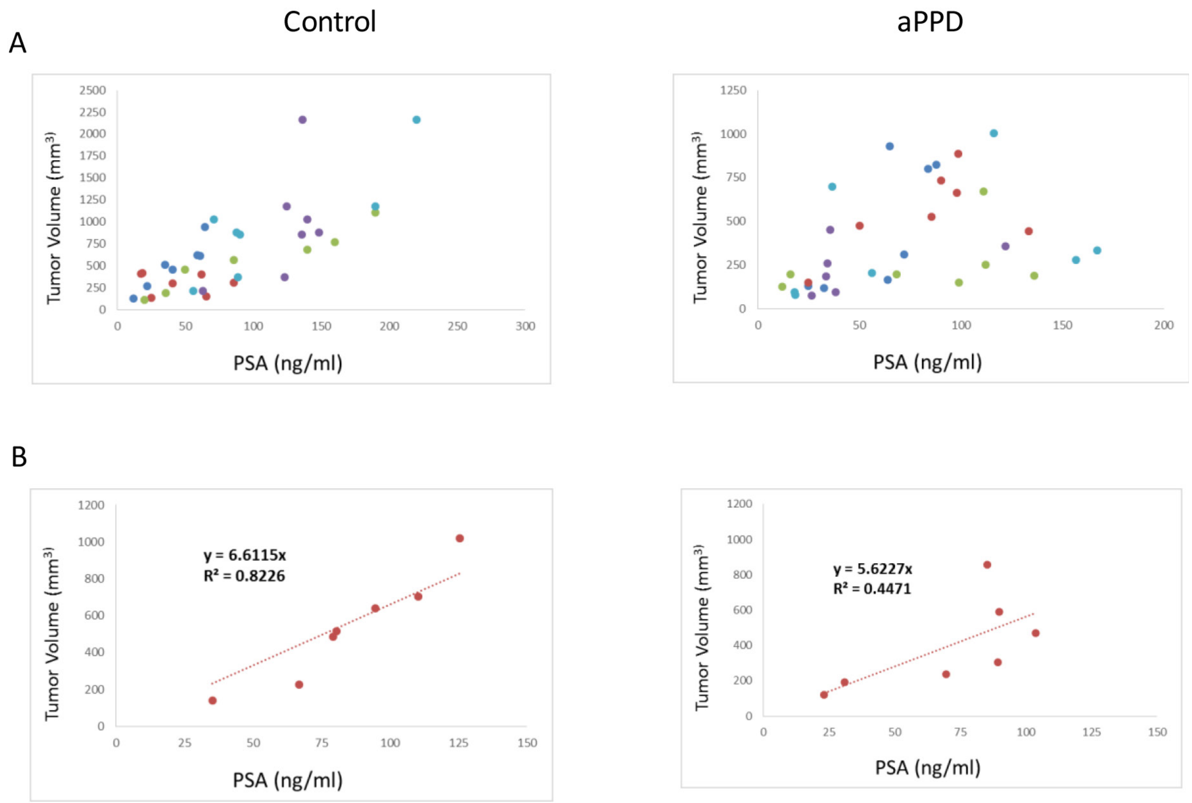

Supplementary Figure 2: Correlation between prostate-specific antigen (PSA) levels with tumor volumes for control and aPPD treated groups. **(Panel A)** scatter plot with raw data (n=8 in each group). **(Panel B)** Pearson's correlation coefficients between mean PSA level with mean tumor volume (n=8).

Supplementary Table 1: Measures of toxicity in C4-2 nude mice serum (n = 3)

Parameters/Units	Control	aPPD
ALB (g/L)	44.5 ± 0.71	47.33 ± 1.53
ALP (U/L)	39 ± 11.31	29 ± 7.55
ALT (U/L)	48 ± 8.48	43.5 ± 10.6
AMY (U/L)	952±54.02	860.5±14.85*
LIP (U/L)	99±36.01	74.67±14.52
TBIL (umol/L)	7 ± 0.05	7.66 ± 3.01
BUN (mmol/L)	5.25 ± 0.77	4.73 ± 0.15
CA (mmol/L)	2.565 ± 0.05	2.77 ± 0.06
PHOS (mmol/L)	2.28 ± 0.08	2.37 ± 0.26
CRE (umol/L)	<18	<18
Glu (mmol/L)	8.7 ± 0.56	9.5 ± 2.58
NA (mmol/L)	154.5 ± 0.71	158 ± 1.73
K (mmol/L)	7.3 ± 0.28	7.7 ± 0.85
TP (g/L)	52.5 ± 0.71	57.33 ± 0.57
GLOB (g/L)	8 ± 0.65	10.33 ± 2.08

Data represents as mean ± SEM.
Abbreviations: Albumin (Alb), Alkaline Phosphatase (ALP), Alanine Aminotransferase (ALT), Amylase (AMY), Lipase (LIP), Blood Urea Nitrogen (BUN), Calcium (Ca), Phosphorus (PHOS), Creatinine (CRE), Glucose (Glu), Sodium (Na), Potassium (K), Total Protein (TP), Globulin (Glb). Gram (g), Litter (L), Mole (mol), Unit (U). A p value < 0.05 was considered significant (*) change compared with control (vehicle-treated group).
